# Community level antibiotic utilization in India and its comparison vis-à-vis European countries: Evidence from pharmaceutical sales data

**DOI:** 10.1371/journal.pone.0204805

**Published:** 2018-10-17

**Authors:** Habib Hasan Farooqui, Sakthivel Selvaraj, Aashna Mehta, David L. Heymann

**Affiliations:** 1 Indian Institute of Public Health—Delhi, Public Health Foundation of India, Gurugram, Haryana, India; 2 Health Economics, Financing and Policy, Public Health Foundation of India, Gurugram, Haryana, India; 3 Faculty of Epidemiology and Population Health, Department of Infectious Disease Epidemiology, London School of Hygiene and Tropical Medicine, London, United Kingdom; Natural Environment Research Council, UNITED KINGDOM

## Abstract

India was the largest consumer of antibiotics in 2010 in the world. Evidence suggests that countries with high per-capita antibiotic consumption have higher rates of antibiotic resistance. To control antibiotic resistance, not only reduction in antibiotic consumption is required, socio-economic factors like access to clean water and sanitation, regulation of private healthcare sector and better governance are equally important. The key objective of this research was to investigate the five year trends in consumption of major antibiotic classes in India and compare them with European Surveillance of Antimicrobial Consumption Network (ESAC-Net) countries. We used Intercontinental Marketing Statistics (IMS) Health (now IQVIA) medicine sales audit data of antibiotic sales in the retail private sector (excluding the hospitals sector) in India. We then standardized dosage trends and assigned defined daily dose (DDD) to all formulations based on the ATC/DDD index. We expressed our data in standardized matrices of DDD per 1000 inhabitants’ per day (DID) to compare antibiotic use in India with ESAC-Net countries. The antibiotic use was plotted and reported by year and antibiotic class. Our main findings are—per capita antibiotic consumption in the retail sector in India has increased from 13.1 DID in 2008 to 16.0 DID in 2012—an increase of ~22%; use of newer class of antibiotics like carbapenems (J01DH), lincosamides (J01FF), glycopeptides (J01XA), 3^rd^ generation cephalosporins (J01DD) and penicillin’s with beta-lactamase inhibitors has risen; and antibiotic consumption rates in India are still low as compared to ESAC-Net countries (16.0 DID vs. 21.54 DID). To conclude our study has provided the first reliable estimates of antibiotic use in the retail sector in India vis-à-vis ESAC-Net countries. In addition, our study could provide a reference point to measure the impact of interventions directed towards reducing antibiotic use.

## Introduction

The burden of infectious diseases in India is among one of the highest in the world. The Million Death Study reported that diseases of infectious origin such as pneumonia and diarrhea accounted for 50% (0·67 million of 1·34 million) of all deaths in children aged 1–59 months in India[1]. The burden of infectious diseases in India is also reflected in both value and volume of antibiotic sales in the country and the size of the antibiotic market as proportion of total pharmaceutical market. Of the total medicines sales worth USD 12.6 Billion in India between 2013 and 2014, anti-infectives contributed around 16.8% of the pharmaceutical market (USD 2.12 Billion)[2]. Van Boeckel et al. reported that India was the largest consumer of antibiotics with 12·9 ×10⁹ units (10·7 units per person) sold in the year 2010. BRICS countries constituted 76% of the overall increase in global antibiotic consumption between 2000 and 2010, of which 23% was attributable to India[3]. Empirical evidence indicates a strong association between antibiotic consumption and subsequent development of bacterial resistance at both individual and community level[4–6]. Goossesn et. al demonstrated that there are higher rates of antibiotic resistance in countries with high per-capita antibiotic consumption in outpatient services through a cross-national database study [5].

In India, several studies have reported increasing resistance to antibiotics[7–9]. The reports on presence and spread of carbapenem (a last resort antibiotic) resistant gram-negative enterobacteriaceae have now put India into the spotlight[10]. Antibiotics are one of the key interventions for prevention and control of infectious diseases and a loss of antimicrobial efficacy is considered a threat to health security[11]. India is especially vulnerable to the loss of antimicrobial efficacy not only because of high treatment costs associated with the resistant infections but also because of limited access to antibiotics. On the other hand, several studies have pointed out that, access to essential medicines in general and access to antibiotics in particular is limited in India [12–16]. Laxminarayan et al reported that under-5 pneumonia deaths are strongly correlated with the availability of antibiotics. In India alone, around 169,760 deaths could potentially be averted through prompt access to effective antibiotics[17]. Hence, antimicrobial surveillance of consumption to understand population-based patterns of antibiotic utilization in India is the need of the hour.

The key objective of this research was to investigate the trends in consumption of major antibiotic classes in India and compare them with European Surveillance of Antimicrobial Consumption Network (ESAC-Net) countries. The imperative of such an approach stems from the fact that for India to reach an ideal level of antibiotic consumption–benchmark needs to be set and compared to. Additionally, the appropriateness of antibiotic consumption is equally a critical parameter in the narrative around antimicrobial resistance. Given that the European nations have reached a stage of near universal access to medicines and also been a well-regulated market, the approach here is to compare Indian consumption pattern to European standards. Although regulation to control antibiotics use exists in India, the experience in India till now is that most medicines including antibiotics can be bought from retail pharmacies without prescription. This is expected to lead to overuse or inappropriate use of antibiotics. Earlier research had reported an increase in antibiotic consumption in India. However, they reported consumption estimates in standard units (SUs) (defined as the smallest dose of formulation–i.e. one tablet or capsule for oral solids, one vial or ampoule for injectable)[3], which did not allow for comparisons with other published studies especially with European Surveillance of Antimicrobial Consumption Network (ESAC-Net) countries. In our study, we have adopted European Surveillance of Antimicrobial Consumption (ESAC) project’s standardized methodology [18] to analyze antibiotic use in India and compared it with ESAC-Net estimates for different classes of antibiotics for the year 2012.

## Materials and methods

### Data source

We relied on a large, nationally representative dataset for antibiotic sales, assembled by IMS Health (now IQVIA). IMS Health is a for-profit agency that collects information on drug sales, health services and technology involving healthcare industry[19]. This study uses national level data on total antibiotic sales (January 2008- December 2012) in the private retail sector collected by IMS Health (now IQVIA). We used the stockist secondary audit (SSA), which captures the sales made from the stockists to the retail pharmacies. The data is collected from a sample of 5,600 stockists across the country and projected to reflect the overall sales in the private retail sector, provides monthly pack-wise sales value and sales volume. Sales volume data include medicine pack details and quantity sold, which are mentioned as standard units (the number of doses sold; IMS identifies a dose as a pill, capsule, or ampoule). The duration of the study was constrained by the availability of sales audit data.

### Measures of antibiotic consumption

We obtained medicine sales audit data of systemic antibiotics grouped according to active substance in the drug, on the basis of Anatomic Therapeutic Chemical (ATC) classification system of the European Pharmaceutical Market Research Association (EphMRA) by IMS Health (now IQVIA). The data was then recoded in accordance with the ATC index (2015) of the World Health Organization Collaborating Centre (WHOCC) for Drug Statistics Methodology. For our study, data on all pharmacological subgroups and chemical subgroups within the group anti-infective was used except antifungal; antibacterial for tuberculosis; and topical antibiotics. We identified 10674 packs of systemic antibacterials, of which 7080 packs were of plain formulations and 3594 packs were of fixed dose combinations (FDCs). DDDs have not been assigned by the WHOCC to a number of formulations marketed in the Indian pharmaceutical market. 66 such packs were identified and excluded from the analysis. Finally, a total of 722 packs were excluded from the analysis due to the problem of missing and ambiguous strengths. This left us with 9886 packs of systemic antibacterials.

For this analysis, we used the Defined Daily Dose (DDD), a widely accepted measure for examining drug utilization coined by the WHOCC for Drug Statistics Methodology. The DDD is the assumed average maintenance dose per day for a drug used for its main indication in adults. We assigned defined daily dose to all the formulations under study based on the ATC/DDD index. We observed that for certain fixed dose combinations (FDCs) of antibiotics, DDDs were not available in WHOCC’s ATC/DDD index. For such molecules, we assigned DDD for the antibacterial to the FDC—if the FDC contained single anti-bacterial and sum of the individual DDD of each anti-bacterial to the FDC—if the FDC contained two or more anti-bacterials[20]. The implementation of the WHO ATC/DDD method enabled us to construct a database for measuring and comparing antimicrobial use. Next, we converted the volumes of anti-bacterials into comparable units by multiplying the standard units (SUs) sold with the strengths and then dividing by defined daily dose (DDD) (i.e. DDDs consumed = (SU*strength)/DDD). To control for the size of the population, we expressed our data in terms of DDD per 1000 inhabitants’ per day (DID). The number of inhabitants in India was based on the mid-year population of the country, which was obtained from the National Population Commission. We analyzed total and proportional antibiotic use expressed in defined daily dose per 1000 inhabitants per day (DID) for the duration of the study (January 2008—December 2012). Antibiotic utilization was plotted and reported by year and antibiotic class. Finally, to compare India’s antibiotic use with ESAC-Net countries through standardized matrices of DID, we relied on ESAC-Net data, which was publicly available at the ATC third and fourth level[21]. We used statistical software STATA 13.0 to perform the analytics.

## Results and discussion

We observed that the systemic antibiotic (J01) use increased from 13.1 DID in 2008 to 16.0 DID in 2012, representing 22.1% increase during the study period (January, 2008 and December, 2012) in India (Table 1). Findings from log linear regression model also indicate that on average utilization of systemic antibiotics in India grew at the rate of 0.5 percent monthly (trend coefficient (β) is 0.005) during the study period (Table A in S1 File).

**Table 1:** Systemic antibiotics (J01) use in India, by antibiotic class, 2008–2012.

	2008	2009	2010	2011	2012
Systemic antibiotics (J01)	DID (%)	DID (%)	DID (%)	DID (%)	DID (%)
Quinolones (J01M)	4.0(30.7)	4.1(28.8)	4.2(27.70	3.9(24.9)	3.7(23.4)
Cephalosporins, carbepenm and monobactum (J01D)	1.7(12.7)	2.0(14.1)	2.3(15.2)	2.5(16.1)	2.6(16.3)
Macrolides and lincosamides (J01F)	1.3(10.1)	1.6(11.1)	1.8(11.8)	1.9(12.4)	2.0(12.3)
Other antibacterials (J01X)	1.6(12.4)	1.8(12.5)	1.9(12.2)	1.9(12.0)	1.9(12.1)
Pencillins (J01C)	1.4 (10.9)	1.6(10.8)	1.7(11.1)	1.8(11.6)	1.9(11.6)
Tetracyclines (J01A)	1.1(8.2)	1.1(7.9)	1.0(6.7)	0.9(5.9)	0.9(5.6)
Combinations of antibacterials (J01RA)	0.5(3.9)	0.5(3.7)	0.6(4.1)	0.7(4.8)	0.9(5.5)
Sulfonamides and trimethoprim, incl. derivatives (J01E)	0.6(4.9)	0.7(4.8)	0.7(4.3)	0.6(3.9)	0.6(3.6)
Streptomycins and aminoglycosides (J01G)	0.4(3.3)	0.5(3.3)	0.4(2.8)	0.4(2.7)	0.4(2.5)
Amphenicols (J01B)	0.1(0.6)	0.1(0.6)	0.1(0.6)	0.1(0.6)	0.1(0.6)
Others	0.3 (2.4)	0.4(2.6)	0.5(3.5)	0.8(5.1)	1.1(6.6)
Total	13.1(100)	14.3(100)	15.4(100)	15.6(100)	16.0(100)

**Fig 1:**
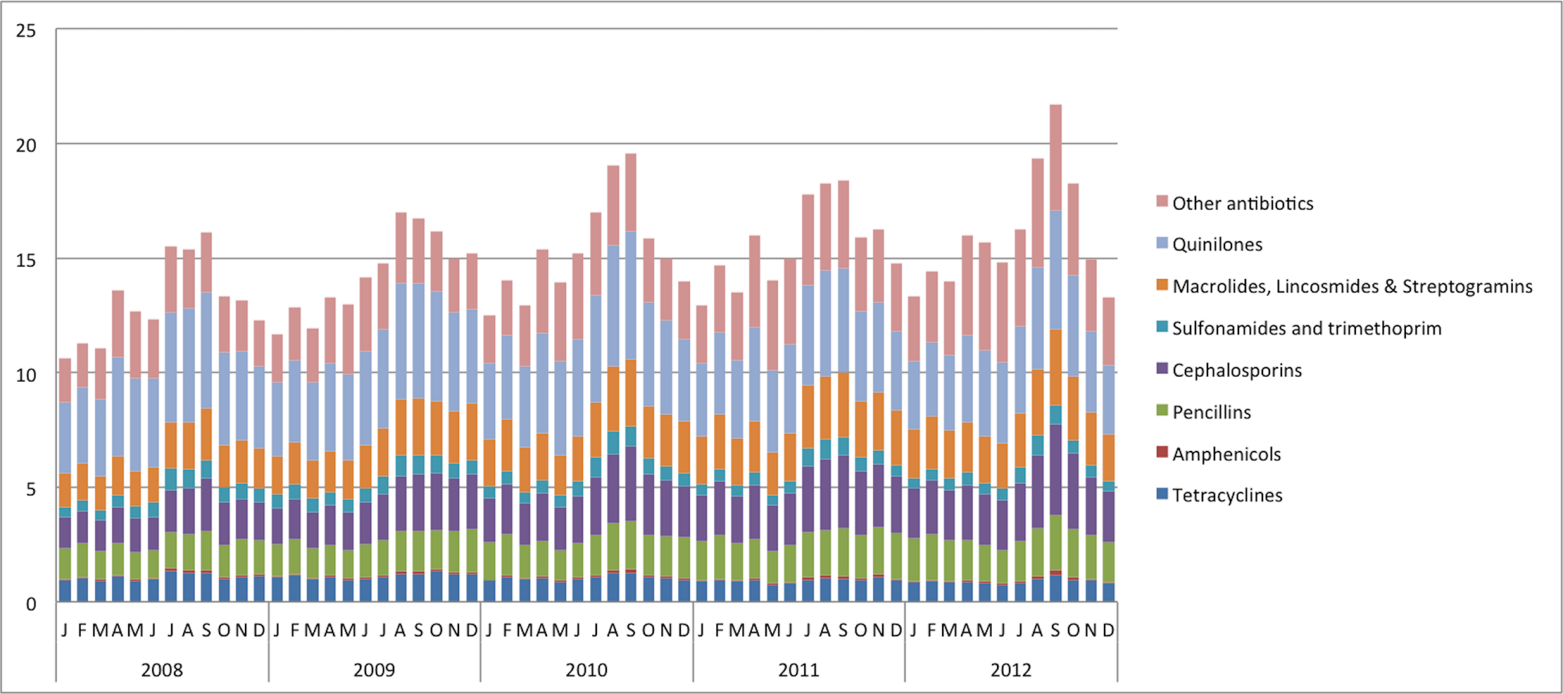
Seasonal variation of systemic antibiotic (J01) use in India, by antibiotic class, 2008–2012.

A disaggregated trend analysis revealed significant variation in consumption across different antibiotic classes. For example, from 2008 to 2012, cephalosporins (J01D) use increased from 1.7 DID to 2.6 DID and proportional use increased from 12.7% to 16.3%. Similarly, macrolides (J01F) use increased from 1.3 DID to 2.0 DID and proportional use increased from 10.1% to 12.3%. However, utilization of quinolones (J01M) declined from 4.0 DID to 3.7 DID and proportional use declined from 30.7% to 23.4%. A declining trend in proportional use was also observed in aminoglycosides (J01G), sulfonamides (J01E) and tetracyclines (J01A). We also observed that antibiotic use was seasonal in nature with peak consumption during September every year except in year 2009 when it was August (Fig 1).

**Fig 2:**
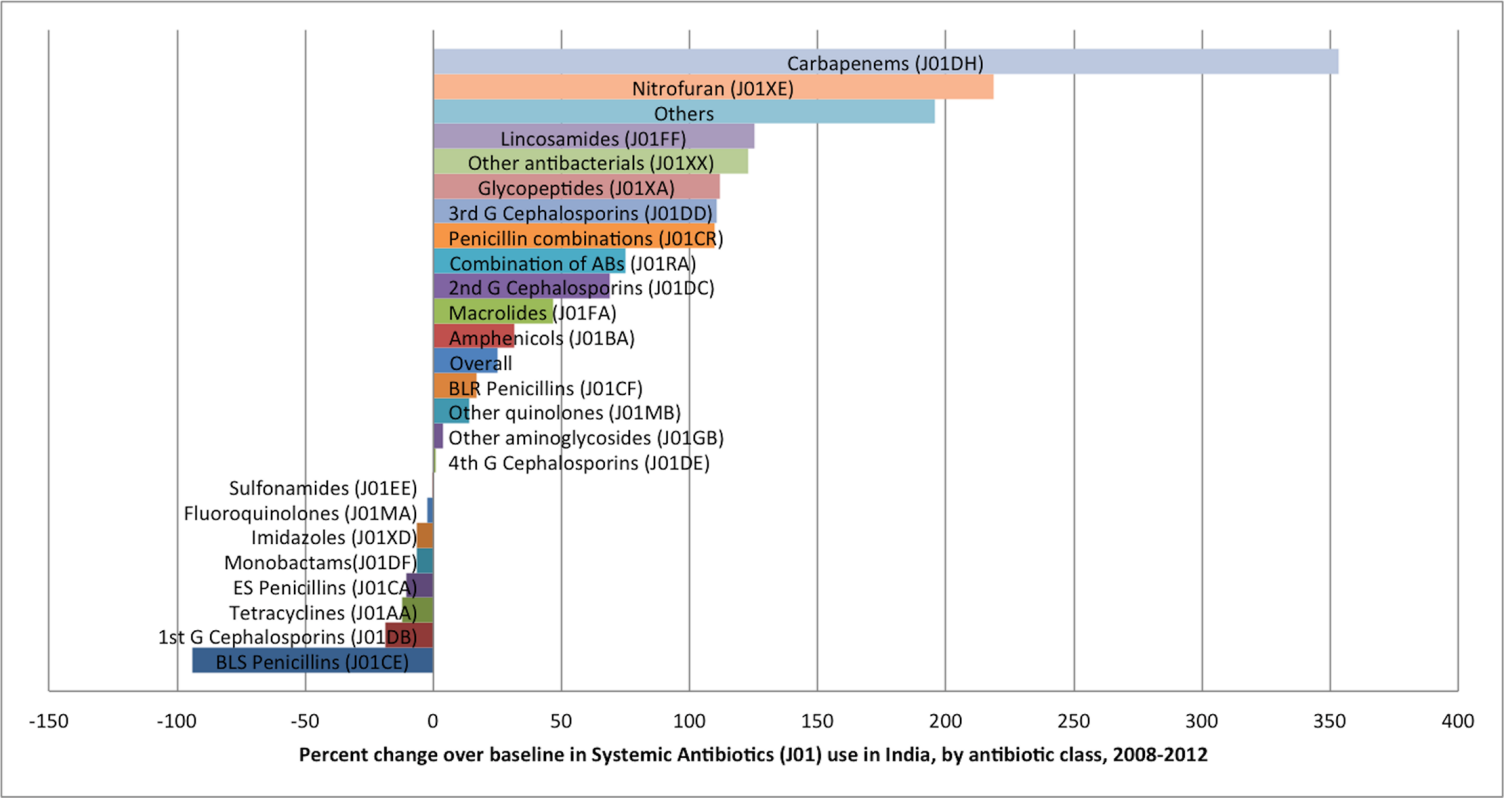
Percent change in systemic antibiotics (J01) use in India, by antibiotic class, 2008–2012.

It may be noted that overall utilization of systemic antibiotics grew a little over 22 percent (Table B in S1 File). However, in disaggregated analysis of antibiotic consumption, we observed significantly high growth for carbapenems (J01DH) (~353 percent) followed by nitrofuran derivatives (J01XE) (~218 percent), lincosamides (J01FF) (~125%), glycopeptides (J01XA) (~111%), third generation cephalosporins (J01DD) (~110%), and penicillin’s with beta-lactamase inhibitors (J01CR)(~109%)(Fig 2).

**Fig 3:**
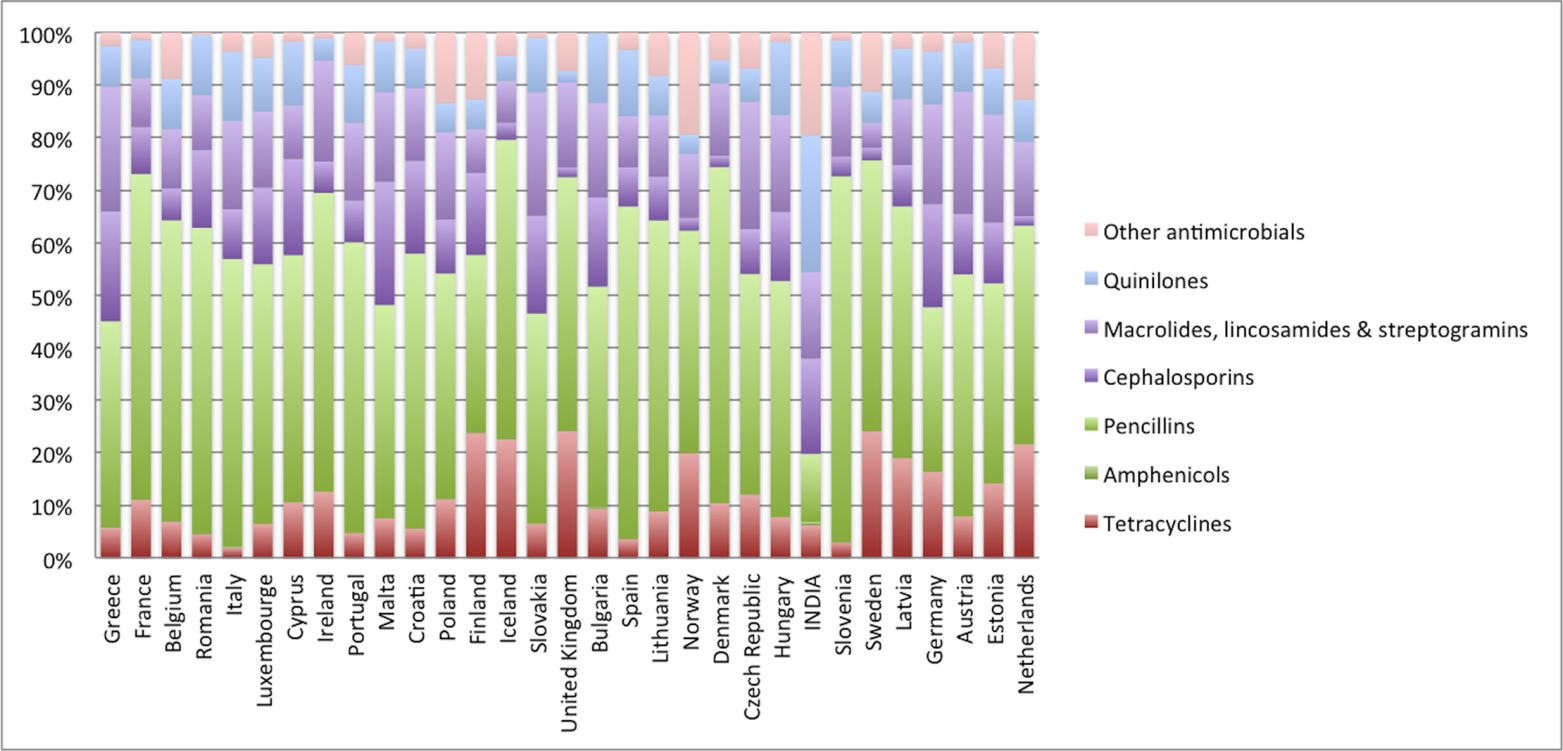
Systemic antibiotic (J01) use in India vis-à-vis ESAC-net countries in 2012.

We also compared systemic antibacterial use (DIDs) in India vis-à-vis ESAC-Net countries at the level of the chemical subgroup (ATC 4) for the year 2012. Fig 3 underscores the heterogeneity of consumption of different antibiotic classes in India vis-à-vis ESAC-Net countries. We observed that the antibiotic consumption (16.0 DID) in India was significantly below the mean ESAC-Net consumption (21.5 DID) in the community sector. Among ESAC-Net countries, highest overall antibiotic consumption was reported for Greece (34.6 DID) and lowest for Netherlands (12.3 DID). Although antibiotic consumption in India was low in comparison to mean ESAC-Net (16.0 DID vs. 21.5 DID), significant differences (in DID terms) were observed among different antibiotic classes. In India, fluoroquinolones (J01MA) had highest proportional antibiotic consumption (3.75 DID; 24.97%) followed by cephalosporins (J01DD) (1.97 DID; 13.15%) and macrolides (J01FA) (1.92 DID; 12.81%). However, in ESAC-Net countries, except for Italy that reported high consumption rates of fluorquinolones (J01MA) and cephalosporins (J01DD) (2.16 DID and 3.81DID, respectively) other countries had much lower consumption rates compared to India (Table C in S1 File). We also observed that mean antibiotic consumption for penicillins combinations including beta-lactamase inhibitors (J01CR), (5.01 DID; 23.27%) extended spectrum penicillins (J01CA) (4.12 DID; 19.11%) and macrolides (J01FA)(2.75 DID; 12.76%) was higher for ESAC-Net countries as compared to India.

To the best of our knowledge, this study for the first time presents standardized antibiotic consumption estimates for India in comparison to ESAC-Net countries. We adopted ESAC project’s standard methods to arrive at antibiotic consumption estimates for India, expressed in DIDs. The key finding points out that per capita antibiotic consumption in India has increased from 13.1 DID in 2008 to 16.0 DID in 2012—an increase of ~22% and increased utilization of newer class of antibiotics such as carbapenems (J01DH), lincosamides (J01FF), glycopeptides (J01XA), third generation cephalosporins (J01DD) and other antibacterials (J01XX). However, antibiotic consumption rates in India are still low as compared to ESAC-Net countries in the community sector (16.0 DID vs. 21.5 DID).

An earlier study on global antibiotic consumption reported that antibiotics consumption in India had increased by 36% between 2000 and 2010[3]. Our findings are consistent with the published evidence; our estimates also suggest a rising trend of antibiotic consumption, ~22% increase from 2008 to 2012. In addition, we also observed that peak antibiotic consumption in month of September every year, consistent with the previous study. India receives the maximum amount of rainfall during this period, poor sanitation and contaminated water in the environment results in a relatively high level of infectious diseases transmission during this period. Chandy et al. have also reported increased fluroquinolones use after monsoon rains in Vellore[22]. There is increased transmission not only of vector borne illnesses like dengue, chikungunya and malaria but also of respiratory and diarrhoeal infections. Various studies have reported inappropriate use of antibiotics in the treatment of viral fever, respiratory and diarrhoeal illnesses even before establishing etiologic diagnosis [23, 24].

The key strength of our study is usage of standardized ESAC-Net methodology for reporting antibiotic consumption in DDD per 1000 inhabitants per day (DIDs). This has enabled us to show that the average antibiotic consumption in India is still lower than majority of ESAC-Net countries. The defined daily dose (DDD) is a universally accepted measurement unit to express drug utilization and to benchmark countries for their level of drug consumption. Monnet et al. reported that the number of DDDs correctly indicate the number of antimicrobial prescriptions for outpatients at the national level[25]. The observed differences in antibiotic use between India and ESAC-Net countries might be explained by variations in social determinants of health, burden of infectious diseases, difference in healthcare system, prescription practices of healthcare providers, and marketing and promotion practices of drug manufactures. For example, Indian health system is dominated by private sector in health-care provision at all levels of care and incomes, which not only includes licensed healthcare professionals but also unlicensed sole practitioners, shops, and medicine vendors [26]. Estimates suggest that of all out-patient and in-patients visits in India, 80% out-patient visits and 62% in-patient visits were made to the private sector[26]. Multiple studies have highlighted that in the private sector[13, 22] newer antibiotics were used more often than the older ones. Kotwani et al. reported, thatmost commonly prescribed cephalosporins (J01D) in the private sector were higher classes of cephalosporins like cefuroxime, cefixime and cefixime plus clavulanic acid[13] suggesting supply driven incentives. Others have argued that private sector not only have supply-side incentives to overprescribe antibiotics but also have less standardized quality assurance system[27–30].

The problem of inappropriate antibiotic use get accentuated many folds because of limited access to healthcare and access to medicines in the public health system[31] which forces patients to seek care in private sector. Limited access to care and medicines in public sector also results into over the counter purchase of antibiotics, which is a major driver of inappropriate use of antibiotics in India. Shet et al. reported that dispensing of antimicrobial drugs without prescription by pharmacies in the private sector in India within an urban setting was unacceptably high (around 67%)[32]. Earlier, Laxminarayanan et al. had reported that nonprescription sales of carbapenems in India are among the highest in the world and contribute to growing carbapenem resistance.[33]A comprehensive literature review demonstrated that misuse of medications in India is widespread and a major public health problem. The determinants were multifactorial including a lack of effective regulation, a lack of education at all levels around appropriate medication use and the risks associated with inappropriate use, as well as an uncoordinated response from the different levels of the health system[34]. However, over the counter access of antibiotics is a complex problem for India on account of the fact that insufficient access and delays in access to antibiotics cause more deaths than antibiotic resistance.[17]

Another key finding of our study is increasing consumption rates of last resort antibiotics such as carbapenems (J01DH), lincosamides (J01FF), glycopeptides (J01XA), third generation cephalosporins (J01DD) and other antibacterials (including linezolid and daptomycin) (J01XX) which belongs to WHO’s watch and reserve group of antibiotics. Our findings are consistent with results reported by other researchers. Using systemic antibiotic sales data of India, Patricia et al. had also reported that sales of key access antibiotic had risen by 20% in India during 2007 to 2012; whereas sales of fixed dose combinations (FDCs) with watch group or reserve group antibiotics had risen more steeply, by 73% and 174%, respectively[35]. Earlier, Van Boeckel et al. had reported increased use of carbapenems and glycopeptides and suggested increasing burden of methicillin-resistant *Staphylococcus aureus* (MRSA) and extended-spectrum β-lactamase (ESBL) producing Gram-negative bacteria[3].

Government of India has initiated a multi-pronged strategy to address the problem of overuse and misuse of antibiotics. For example, Schedule H1 has been implemented by the Central Drugs Standard Control Organization (CDSCO) to prevent over-the-counter (OTC) sales of important antibiotics, such as third and fourth-generation cephalosporins, carbapenems, antituberculosis drugs and newer fluoroquinolones[36]. Under this new Schedule H1, the sale of these drugs without a doctor’s prescription would attract substantial penalties. Similarly, National Center for Disease Control (NCDC) has developed national treatment guidelines for antimicrobial use in infectious diseases to guide healthcare providers on appropriate use of antibiotics[37]. Other initiatives include Mission Indradhanush[38] for improving access to vaccines and to potentially reduce need for antibiotics. However, potential impact of these interventions in reducing antibiotic use is yet to be determined.

Evidence from the National Health Accounts for India demonstrated that during 2013–14, an estimated INR 1331 per capita was spent on medicines, out of which households alone incurred INR 1200 per capita, suggesting 90% of all medicines spending in the country is in retail segment[39]. This reliance on direct out-of-pocket payments, for financing and access to healthcare has resulted into a complex situation where on one hand–consumption of newer generation of antibiotics has increased significantly and on the other hand—total antibiotic use is still low, highlighting potential access to medicine issues. We argue that Government intervention directed towards universal health coverage and free medicine initiatives could ameliorate this situation by deterring patients from seeking healthcare from unlicensed practitioners in the private sector[27]. In addition, capacitiy building of the public sector in infectious diseases management to diagnose, prescribe, and provide appropriate high-quality antibiotics are required.

Our analysis was based on stockist secondary audits, which represent sales made by the stockists to the retailers and do not capture sales made to the hospitals and doctors. Our study does not account for the utilization of antibiotics in the public sector. We suspect that this may have biased our antibiotic consumption estimates downwards, albeit marginally, as the public sector contribution to the overall drug consumption at national level is less than 10%, in value terms[39].

## Conclusions

Our study has provided first reliable estimates of antibiotic use in India vis-à-vis ESAC-Net countries. Interventions directed towards achieving Universal Health Coverage, particularly free access to medicines through public health facilities should be implemented to mitigate demand for antibiotics in the private sector. Since antibiotic use is both prescription and self-medication driven in India, education campaigns and behavior change communication strategies to address overuse and misuse of antibiotics are required. Specific regulation with reference to licensing, sales and prescription of antibiotics are in force, their effectiveness has not been evaluated formally; additional research is required to address this knowledge gap.

## Supporting information

S1 File Supporting file. (PDF)
