# Supplementary material for: Community level antibiotic utilization in India and its comparison vis-à-vis European countries: Evidence from pharmaceutical sales data
Source: PLoS One. 2018 Oct 17;13(10):e0204805. doi: 10.1371/journal.pone.0204805 (PMC6192587; doi:10.1371/journal.pone.0204805)
Supplement: S1 File — (PDF) [file pone.0204805.s001.pdf]

# Supporting File.

The log linear trend for assessing utilization of systemic antibacterials (see equation below)

$ln(u_t) = \alpha + \beta t + \epsilon_t$

dependent variable - $\ln(u_t)$ - is the utilization at time t measured in terms of DDD/1000population/day,

independent variable - t - is the time,

α and β - are the intercept and the slope respectively,

$\epsilon_t$ - is the error term.

Table A. Log linear regression analysis results for total systemic antibiotic utilization in India (2008 to 2012)

| Variable | Coefficients(β) | 95% conf. interval | |
|---|---|---|---|
| Time (t) | 0.005<br>(0.0009)<br>*** | 0.003 | 0.007 |
| _Cons. | 2.546<br>(0.3278)<br>*** | 2.481 | 2.612 |
| *R squared = 0.30 and Adj. R squared = 0.29*<br>*Durbin-Watson d-statistic (2, 60) = .78* | | | |

Note: standard errors are in parentheses; *** significance at $p<0.05$, ** significance at $p<0.1$, * significance at $p<0.3$

Table B. Percent change over baseline in Systemic Antibiotics (J01) use in India, by antibiotic class, 2008-2012

| | Month 1 | Month 60 | Growth (month 60 over month 1) |
|---|---|---|---|
| Tetracyclines (J01AA) | 0.94 | 0.83 | -12.12 |
| Amphenicols (J01BA) | 0.03 | 0.04 | 31.55 |
| Extended spectrum Penicillins (J01CA) | 0.98 | 0.87 | -10.59 |
| Beta-lactamase sensitive penicillins (J01CE) | 0.01 | 0.0004 | -94.11 |
| Beta-lactamase resistant penicillins (J01CF) | 0.002 | 0.002 | 16.71 |
| Penicillin combinations incl. beta-lactamase inhibitors (J01CR) | 0.40 | 0.84 | 109.82 |
| First-generation cephalosporins (J01DB) | 0.42 | 0.34 | -18.93 |
| Second-generation cephalosporins (J01DC) | 0.14 | 0.24 | 68.98 |
| Third-generation cephalosporins (J01DD) | 0.78 | 1.65 | 110.76 |
| Fourth-generation cephalosporins (J01DE) | 0.0004 | 0.0004 | 0.90 |
| Monobactams(J01DF) | 0.00002 | 0.00002 | -6.58 |
| Carbapenems (J01DH) | 0.0001 | 0.001 | 353.21 |
| Sulfonamides and trimethoprim, incl. derivatives (J01EE) | 0.44 | 0.43 | -0.27 |
| Macrolides (J01FA) | 1.19 | 1.75 | 46.75 |
| Lincosamides (J01FF) | 0.02 | 0.04 | 125.18 |
| Other aminoglycosides (J01GB) | 0.27 | 0.28 | 3.94 |

| | | | |
|---|---|---|---|
| Fluoroquinolones (J01MA) | 3.08 | 3.00 | -2.58 |
| Other quinolones (J01MB) | 0.01 | 0.01 | 13.86 |
| Combinations of antibacterials (J01RA) | 0.38 | 0.67 | 74.91 |
| Glycopeptide antibacterials (J01XA) | 0.0003 | 0.001 | 111.75 |
| Polymyxins (J01XB) | 0.00 | 0.00001 | |
| Imidazole derivatives (J01XD) | 1.05 | 0.98 | -6.53 |
| Nitrofuran derivatives (J01XE) | 0.02 | 0.05 | 218.49 |
| Other antibacterials (J01XX) | 0.15 | 0.33 | 122.90 |
| Others | 0.30 | 0.90 | 195.69 |
| | 10.61 | 13.27 | 25.02 |

Table C. Total Systemic Antibiotics (J01) use in India and ESAC-net countries, by antibiotic class, 2012

| | DID (%) | | Range of use in ESAC-Net | |
|---|---|---|---|---|
| | INDIA | ESAC-Net (mean) | Highest DID (country) | Lowest DID (country) |
| Systemic Antibiotics (J01) | 16.0 (100) | 21.5 (100) | 34.6(Greece) | 12.3(Netherland) |
| Tetracyclines (J01AA) | 0.89 (5.94) | 2.22 (10.3) | 4.92 (Greece) | 0.89(Netherland |
| Amphenicols (J01BA) | 0.09 (0.60) | 0.01 (0.03) | 0.06 (Italy) | <0.0001(Greece) |
| Extended | 1.00 (6.67) | 4.12 (19.1) | 10.1 (France) | 1.00 (Malta) |

| | | | | |
|---|---|---|---|---|
| spectrum Penicillins (J01CA) | | | | |
| Beta-lactamase sensitive penicillins (J01CE) | 0.00 (0.01) | 0.97 (4.52) | 4.77 (Denmark) | <0.02 (Latvia) |
| Beta-lactamase resistant penicillins (J01CF) | 0.00 (0.02) | 0.38 (1.78) | 1.82 (Sweden) | <0.01 (Greece) |
| Penicillin combinations incl. beta-lactamase inhibitors (J01CR) | 0.87 (5.78) | 5.01 (23.3) | 11.7 (Italy) | 0.3 (Sweden) |
| First-generation cephalosporins (J01DB) | 0.38 (2.53) | 0.31 (1.44) | 2.42 (Finland) | <0.01 (Denmark) |
| Second-generation cephalosporins (J01DC) | 0.26 (1.77) | 1.57 (7.27) | 6.78 (Greece) | 0.02 (Sweden) |
| Third-generation | 1.97 (13.2) | 0.43 (1.99) | 2.16(Italy) | <0.01(Poland) |

| | | | | |
|---|---|---|---|---|
| cephalosporins (J01DD) | | | | |
| Fourth-generation cephalosporins (J01DE) | 0.00 (0.0) | 0.01 (0.03) | 0.03 (Greece) | <0.01 (Luxembourg) |
| Carbapenems (J01DH) | 0.00 (0.00) | 0.04 (0.19) | 0.16 (Greece) | <0.01 (Germany) |
| Sulfonamides and trimethoprim, incl. derivatives (J01EE) | 0.58 (3.87) | 0.48 (2.24) | 1.45(Poland) | <0.08(Austria) |
| Macrolides (J01FA) | 1.92 (12.8) | 2.75 (12.8) | 7.71 (Greece) | 0.33 (Sweden) |
| Lincosamides (J01FF) | 0.05 (0.31) | 0.28 (1.29) | 0.76 (Germany) | <0.03 (UK) |
| Streptomycins (J01GA) | 0.02 (0.15) | 0.00 (0.02) | 0.06 (Romania) | <0.0003 (Spain) |
| Other aminoglycosides (J01GB) | 0.38 (2.54) | 0.10 (0.47) | 0.59 (Czech Rep) | <0.008 (Spain) |
| Fluoroquinolones (J01MA) | 3.75 (25.0) | 1.82 (8.43) | 3.81(Italy) | 0.42 (UK) |
| Other quinolones (J01MB) | 0.01 (0.09) | 0.01 (0.05) | 0.11 (Italy) | <0.009(Netherland) |

| | | | | |
|---|---|---|---|---|
| Combinations of antibacterials (J01RA) | 0.88 (5.85) | 0.01 (0.04) | 0.21 (Spain) | NA |
| Glycopeptide antibacterials (J01XA) | 0.00 (0.00) | 0.03 (0.12) | 0.1 (Greece) | <0.001 (Poland) |
| Polymyxins (J01XB) | 0.00 (0.00) | 0.04 (0.18) | 0.6 (UK) | <0.003 (Poland) |
| Imidazole derivatives (J01XD) | 1.54 (10.2) | 0.07 (0.33) | 0.34 (Estonia) | <0.001 (Spain) |
| Nitrofuran derivatives (J01XE) | 0.06 (0.39) | 0.63 (2.93) | 2.87 (Poland) | <0.02 (Irelnd) |
| Other antibacterials (J01XX) | 0.34 (2.29) | 0.31 (1.44) | 3.0 (Finland) | 0.01 (Romania) |

#Antibiotic classes with consumption >0.01 DID are reported
